# Genome Sequences of Rhinovirus A Isolates from Wisconsin Pediatric Respiratory Studies

**DOI:** 10.1128/genomeA.00200-14

**Published:** 2014-03-27

**Authors:** Stephen B. Liggett, Yury A. Bochkov, Tressa Pappas, Robert F. Lemanske, James E. Gern, Naomi Sengamalay, Xuechu Zhao, Qi Su, Claire M. Fraser, Ann C. Palmenberg

**Affiliations:** a Department of Medicine, University of South Florida Morsani College of Medicine, Tampa, Florida, USA; b Department of Pediatrics, University of Wisconsin School of Medicine and Public Health, Madison, Wisconsin, USA; c Institute for Genome Sciences, University of Maryland School of Medicine, Baltimore, Maryland, USA; d Institute for Molecular Virology, University of Wisconsin-Madison, Madison, Wisconsin, USA

## Abstract

Full-length or nearly full-length RNA genome sequences for 98 rhinovirus (RV) A isolates (from the *Enterovirus* genus of the *Picornaviridae* family), representing 43 different genotypes, were resolved as part of ongoing studies to define RV genetic diversity and its potential link to respiratory disease.

## GENOME ANNOUNCEMENT

The University of Wisconsin hospitals and clinics in Madison, WI, are actively engaged in virus surveillance as part of several long-term studies to determine the extent of diversity of rhinovirus (RV) genomes in respiratory secretions and to ascertain whether virus genomic sequence variation is linked to cold symptoms and asthma exacerbations. Respiratory samples from infant cohorts of the Childhood Origins of Asthma (COAST), RhinoGen, and T Regulatory Cells and Childhood Asthma (T-Reg) studies were surveyed for these data. The initial screens employed multiplex PCR assays (1), rhinovirus PCR (2), or both. Of samples collected between 1999 and 2010, hundreds were identified as solitary RV infections. Partial sequencing of the 5′-untranslated region (UTR) assigned many initial species (A, B, or C) and genotype identifications (GenBank accession no. JX041186 to JX041253), as has been reported (3). Nearly complete-genome-length datasets were then derived using massively parallel sequencing applied directly to patient samples without viral propagation, thereby eliminating the influence of amplification pressures.

The frozen nasal lavage fluids (300 µl) were treated with DNase 1 (Ambion) and the RNA was extracted using Xpress EZ-RNA kits (Express Biotech). cDNA was prepared using the Ovation RNA-Seq system (NuGEN), and purified using Agencourt RNAClean XP beads (Agencourt). Libraries were prepared from these (1.5 µg) using TruSeq DNA kits (Illumina). Adapters containing 6-nucleotide index sequences were ligated to the fragments of the libraries. After size selection and PCR enrichment, the indexed library pools were sequenced on 2 × 100 bp runs using a HiSeq 2000 sequencer (Illumina). Potential RV reads were first identified using low-stringency BLAST searches against all known RV sequences (4). These were assembled using the Velvet software (Illumina). This single-pass methodology gave, on average, 93% coverage to a depth of 8 to 10 reads for 179 study-specific isolates.

The preferred RV nomenclature (5) designates the species letter (A, B, or C) and type number (e.g., A16). The clinical isolate designations are unique to each accession number. With regard to RV-A, the most populous species (78 genotypes), the sequencing resolved full-length or nearly full-length genomes for 98 isolates, representing 43 different genotypes. These include the first descriptions of new genotypes A103 to A106. Of these, A104 to A106 had been prematurely assigned as A24, A57, and A71, respectively (3). Relative to the prototype RV-A genomes, which average 7,132 bases (b) (4), most of the new assemblies were missing the difficult-to-sequence 5′ and/or 3′ termini (average, Δ379 b) and occasionally, short internal fragments (<100 b) for which the contigs could not be explicitly linked. Nevertheless, every new sequence (average, 6,686 b; median, 6,753 b) was unambiguously aligned with the complete index compilation of the RV prototype genomes (4) for full genotyping according to the recognized International Committee on Taxonomy of Viruses (ICTV) criteria for this species (5). This collection represents the largest group of RV-A data at this level of sequence coverage derived directly from clinical samples.

### Nucleotide sequence accession numbers.

Each contiguous RV-A data set has been deposited in GenBank using the listed accession numbers. Each unit described here is the first genome version of the sequence of that isolate: A01, JN798558, JN815255, JN837694, and JQ837724; A10, JN541269, JN798575, JN798582, and JN815247; A12, JF781511; A15, JN541268; A16, JN562722, JN614992, JN798564, JN798574, JN815253, JN990704, and JX074057; A18, JF781496 and JF781508; A19, JQ747746 and JQ747750; A20, JN541270, JN614993, JN798571, and JQ994494; A21, JN837693 and JQ747747; A23, JN621244, JN815254, and JN837696; A24, JN798563; A28, JN798577, JN798580, and JQ747751; A30, JN798557; A33, JN815250 and JN990707; A34, JF781510, JF781512, and JN562720; A36, JF781497, JN614994, JN621243, JN798583, JN798584, JN815241, JN815242, JN815246, JN837697, and JX074050; A38, JN541272 and JQ994496; A40, JN798579, JQ245967, and JX074051; A43, JN815237; A44, JN815252; A47, JN837692; A49, JN621241, JN798561, and JN798589; A51, JN562725; A53, JN798587 and JQ837718; A58, JX025558; A59, JN541266; A60, JN798590; A61, JN798560; A65, JF781504 and JQ245966; A66, JN112340, JN621246, and JQ837715; A67, JN621245; A68, JN798578; A75, JF781503 and JN837690; A76, JN815238, JX074049, and JX074055; A80, JN798576, JN798586, and JN990705; A82, JN798556, JN798585, and JQ837722; A89, JQ837716 and JQ837719; A101, JQ245965; A103, JQ747749 and JQ994499; A104, JN562727, JX074047, and JX193797; A105, JN614995 and JN990699; A106, JQ245971 and JX025555.
